# Complete coding genome sequence of a *Teschovirus A* genotype strain

**DOI:** 10.1128/mra.00063-24

**Published:** 2024-02-13

**Authors:** Yanbin Ding, Xiaofang Xie, Jiefeng Huang, Can Yin, Taotao Yang

**Affiliations:** 1 College of Life Sciences and Resource Environment, Yichun University, Yichun, Jiangxi, China; 2 Yiyang Vocational and Technical College, Yiyang, Hunan, China; 3 Loudi Municipal Agriculture and Rural Affairs Bureau, Loudi, Hunan, China; 4 Huaihua Vocational and Technical College, Huaihua, Hunan, China; Department of Biology, Queens College, Queens, Texas, USA

**Keywords:** *Teschovirus*, genotype

## Abstract

This study reports the complete coding genome sequence of a novel *Teschovirus A* genotype strain, SG2, isolated from the fecal sample of an infected indigenous pig in western Jiangxi, China.

## ANNOUNCEMENT

Porcine teschovirus (PTV) causes poliencephalomyelitis in pigs (1). Belonging to the *Picornaviridae* family, *Teschovirus* genus, and comprising two species, *Teschovirus A* and *Teschovirus B*, PTV genomes consist of a single positive-stranded RNA whose classification is based on sequencing of complete coding genome, also known as polyprotein genome (2). The International Committee on Taxonomy of Viruses (ICTV) described 19 PTV genotypes (*Teschovirus A*: teschovirus A1 to -A14, *Teschovirus B*: teschovirus B1 to -B3; recombinant viruses: teschovirus A15_CP_-B_Pol_, teschovirus A16 _CP_-B_Pol_) in latest published *Teschovirus* taxonomy information (3). Here, we report the complete coding genome sequence for reference strain SG2, a putative novel *Teschovirus A* genotype.

SG2 was isolated from the PCR-positive fecal sample of an infected indigenous pig in Yichun village (western Jiangxi, China) in March 2019. The supernatants of the homogenized fecal sample were incubated with porcine kidney (PK-15) cells. Cultures were freeze-thawed three times and centrifuged at 4,000 × *g* for 5 min. The clarified supernatants were then passaged two times in fresh PK-15 cells. The viral stocks of SG2 strain propagated in PK-15 cell monolayers were used for RNA extraction with FastPure Viral DNA/RNA Mini Kit (Vazyme, Nanjing, China) and cDNA synthesis with RevertAid First Strand cDNA Synthesis Kit (Thermo Scientific, Waltham, MA, USA). First, SG2 cDNA was identified as PTV using a previously developed qRT-PCR method (4). Then, five primer sets designed from our previous work (5) were used to amplify overlapping regions of the complete coding genome sequence of the SG2 strain. The PCR products were purified and then Sanger sequenced (BioSune, Shanghai, China). In all, 12 sequencing reads of about 900 bp with overlapped at least 30 match sizes were obtained. The sequenced results were trimmed by the SeqMan program using a minimum match percentage of 80% within DNAStar software (6).

**Fig 1:**
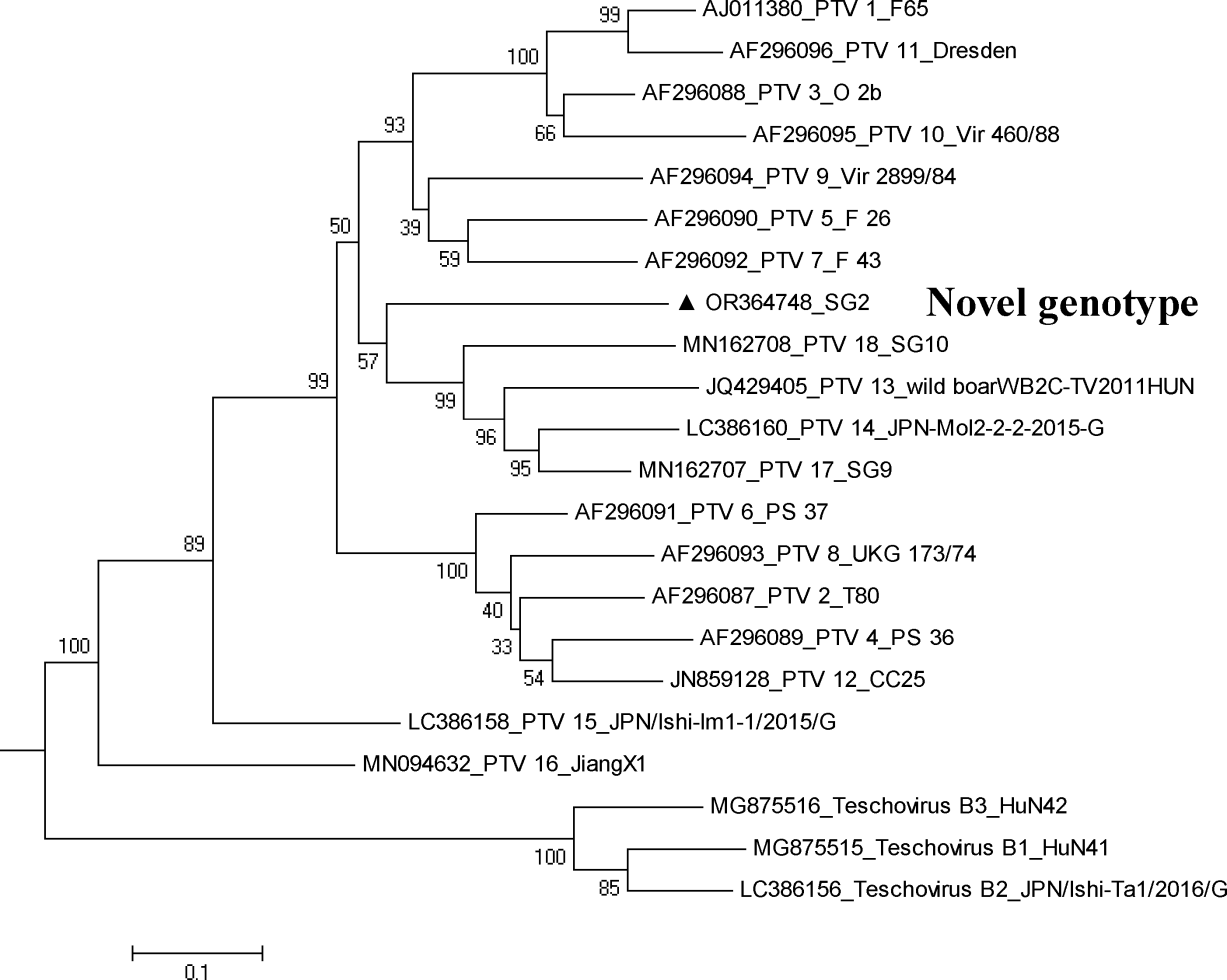
Phylogenetic tree based on the amino acid (aa) sequences of the P1 gene. The sequences were aligned by the MUSCLE program within MEGA 6.06 software (7). The tree was constructed using the maximum likelihood in the Jones-Taylor-Thornton model with gamma-distributed rates and proportions of invariant sites (G + I) in MEGA 6.06 software (bootstrap test of 1,000 replicates) (7). The scale bar indicates aa substitutions per site. The reference sequences were collected from the NCBI database. The strain isolated in the present study is indicated by a triangle.

The near complete genome of 6,815 nucleotides (44.67% G-C content) containing a complete polyprotein coding sequence was obtained. Alignment of the coding sequences of the SG2 strain with those of PTV reference strains (Table 1) revealed that the SG2 strain contains a polyprotein coding genome with 6,627 nucleotides, encoding a polyprotein with 2,209 amino acids. Identity analyses of the polyprotein coding genome sequence of SG2 strain with those of *Teschovirus A* genotypes reference strains using MegAlign program within DNAStar software (6) revealed higher deduced aa sequence homology of 87.6 to 90.4%, while showed lower aa homology (76.1 to 76.4%) to the *Teschovirus B* genotypes. A similar result was obtained when analyzing P1 and 2C + 3 CD genes. These results verify SG2 strain belonged to *Teschovirus A*. To further confirm the genotype of the SG2 strain, the phylogenetic tree of P1 (structural protein coding region, commonly used for PTV genotyping) was constructed using the ML method within MEGA 6.06 software (7). The topological structure of the phylogenetic tree revealed the SG2 strain was found in a separate branch distinct from the other known PTV genotype strains, indicating SG2 strain belonged to a novel PTV genotype (Fig. 1).

**TABLE 1:** Porcine teschovirus reference strains used in the study

Virus isolate	Genotype	Collection date	GenBank accession No.	Geographic origin	Sequence length
F65	1	1967	AJ011380	UK	7117
T80	2	1958	AF296087	UK	7017
O 2b	3	1965	AF296088	USA	7012
PS 36	4	1965	AF296089	USA	7014
F 26	5	1958	AF296090	USA	7008
PS 37	6	1965	AF296091	USA	7018
F 43	7	1958	AF296092	Germany	7014
UKG 173/74	8	1974	AF296093	UK	7017
Vir 2899/84	9	1984	AF296094	Germany	7006
Vir 460/88	10	1988	AF296095	Germany	7009
Dresden	11	1965	AF296096	Germany	7111
CC25	12	2006	JN859128	Spain	6952
Wild boar/WB2C-TV/2011/HUN	13	2011	JQ429405	Hungary	7123
JPN/MoI2-2-2/2015/G	14	2015	LC386160	Japan	3342
JPN/Ishi-Im1-1/2015/G	15	2015	LC386158	Japan	6820
JiangX1	16	2019	MN094632	China	6799
SG9	17	2019	MN162707	China	6773
SG10	18	2019	MN162708	China	6776
HuN41	B1	2017	MG875515	China	6931
JPN/Ishi-Ta1/2016/G	B2	2016	LC386156	Japan	7163
HuN42	B3	2017	MG875516	China	6922

In the present study, a putative novel *Teschovirus A* genotype was identified. The novel genotype sequence will enrich the knowledge of *Teschovirus* taxonomy.

## Data Availability

The complete coding genome sequence of SG2 is available at GenBank under the accession number OR364748. Raw sequence data were deposited in the SRA under BioProject number PRJNA1066424 and BioSample number SAMN39488039.
